# Risk of Cardiovascular Disease and Total Mortality in Adults with Type 1 Diabetes: Scottish Registry Linkage Study

**DOI:** 10.1371/journal.pmed.1001321

**Published:** 2012-10-02

**Authors:** Shona J. Livingstone, Helen C. Looker, Eleanor J. Hothersall, Sarah H. Wild, Robert S. Lindsay, John Chalmers, Stephen Cleland, Graham P. Leese, John McKnight, Andrew D. Morris, Donald W. M. Pearson, Norman R. Peden, John R. Petrie, Sam Philip, Naveed Sattar, Frank Sullivan, Helen M. Colhoun

**Affiliations:** 1 University of Dundee, Dundee, United Kingdom; 2 University of Edinburgh, Edinburgh, United Kingdom; 3 University of Glasgow, Glasgow, United Kingdom; 4 National Health Service (NHS) Fife, Kirkcaldy, United Kingdom; 5 NHS Greater Glasgow, Glasgow, United Kingdom; 6 NHS Lothian, Edinburgh, United Kingdom; 7 University of Aberdeen, Aberdeen, United Kingdom; 8 NHS Forth Valley, Falkirk, United Kingdom; Yale University, United States of America

## Abstract

**Background:**

Randomized controlled trials have shown the importance of tight glucose control in type 1 diabetes (T1DM), but few recent studies have evaluated the risk of cardiovascular disease (CVD) and all-cause mortality among adults with T1DM. We evaluated these risks in adults with T1DM compared with the non-diabetic population in a nationwide study from Scotland and examined control of CVD risk factors in those with T1DM.

**Methods and Findings:**

The Scottish Care Information-Diabetes Collaboration database was used to identify all people registered with T1DM and aged ≥20 years in 2005–2007 and to provide risk factor data. Major CVD events and deaths were obtained from the national hospital admissions database and death register. The age-adjusted incidence rate ratio (IRR) for CVD and mortality in T1DM (*n* = 21,789) versus the non-diabetic population (3.96 million) was estimated using Poisson regression. The age-adjusted IRR for first CVD event associated with T1DM versus the non-diabetic population was higher in women (3.0: 95% CI 2.4–3.8, *p*<0.001) than men (2.3: 2.0–2.7, *p*<0.001) while the IRR for all-cause mortality associated with T1DM was comparable at 2.6 (2.2–3.0, *p*<0.001) in men and 2.7 (2.2–3.4, *p*<0.001) in women. Between 2005–2007, among individuals with T1DM, 34 of 123 deaths among 10,173 who were <40 years and 37 of 907 deaths among 12,739 who were ≥40 years had an underlying cause of death of coma or diabetic ketoacidosis. Among individuals 60–69 years, approximately three extra deaths per 100 per year occurred among men with T1DM (28.51/1,000 person years at risk), and two per 100 per year for women (17.99/1,000 person years at risk). 28% of those with T1DM were current smokers, 13% achieved target HbA_1c_ of <7% and 37% had very poor (≥9%) glycaemic control. Among those aged ≥40, 37% had blood pressures above even conservative targets (≥140/90 mmHg) and 39% of those ≥40 years were not on a statin. Although many of these risk factors were comparable to those previously reported in other developed countries, CVD and mortality rates may not be generalizable to other countries. Limitations included lack of information on the specific insulin therapy used.

**Conclusions:**

Although the relative risks for CVD and total mortality associated with T1DM in this population have declined relative to earlier studies, T1DM continues to be associated with higher CVD and death rates than the non-diabetic population. Risk factor management should be improved to further reduce risk but better treatment approaches for achieving good glycaemic control are badly needed.

*Please see later in the article for the Editors' Summary*

## Abstract 2

Helen Colhoun and colleagues report findings from a Scottish registry linkage study regarding contemporary risks for cardiovascular events and all-cause mortality among individuals diagnosed with type 1 diabetes.

## Editors' Summary

**Background.** People with diabetes are more likely to have cardiovascular disease such as heart attacks and strokes. They also have a higher risk of dying prematurely from any cause. Controlling blood sugar (glucose), blood pressure, and cholesterol can help reduce these risks. Some people with type 1 diabetes can achieve tight blood glucose control through a strict regimen that includes a carefully calculated diet, frequent physical activity, regular blood glucose testing several times a day, and multiple daily doses of insulin. Other drugs can reduce blood pressure and cholesterol levels. Keeping one's weight in the normal range and not smoking are important ways in which all people, including those with type 1 diabetes can reduce their risks of heart disease and premature death.

**Why Was This Study Done?** Researchers and doctors have known for almost two decades what patients with type 1 diabetes can do to minimize the complications from the disease and thereby reduce their risks for cardiovascular disease and early death. So for some time now, patients should have been treated and counseled accordingly. This study was done to evaluate the current risks for have cardiovascular disease and premature death amongst people living with type 1 diabetes in a high-income country (Scotland).

**What Did the Researchers Do and Find?** From a national register of all people with type 1 diabetes in Scotland, the researchers selected those who were older than 20 years and alive at any time from January 2005 to May 2008. This included about 19,000 people who had been diagnosed with type 1 diabetes before 2005. Another 2,600 were diagnosed between 2005 and 2008. They also obtained data on heart attacks and strokes in these patients from hospital records and on deaths from the natural death register. To obtain a good picture of the current relative risks, they compared the patients with type 1 diabetes with the non-diabetic general Scottish population with regard to the risk of heart attacks/strokes and death from all causes. They also collected information on how well the people with diabetes controlled their blood glucose, on their weight, and whether they smoked.

They found that the current risks compared with the general Scottish population are quite a bit lower than those of people with type 1 diabetes in earlier decades. However, people with type 1 diabetes in Scotland still have much higher (more than twice) the risk of heart attacks, strokes, or premature death than the general population. Moreover, the researchers found a high number of deaths in younger people with diabetes from coma—caused by either too much blood sugar (hyperglycemia) or too little (hypoglycemia). Severe hyperglycemia and hypoglycemia happen when blood glucose control is poor. When the scientists looked at test results for HbA_1c_ levels (a test that is done once or twice a year to see how well patients controlled their blood sugar over the previous 3 months) for all patients, they found that the majority of them did not come close to controlling their blood glucose within the recommended range.

When the researchers compared body mass index (a measure of weight that takes height into account) and smoking between the people with type 1 diabetes and the general population, they found similar proportions of smokers and overweight or obese people.

**What Do these Findings Mean?** The results represent a snapshot of the recent situation regarding complications from type 1 diabetes in the Scottish population. The results suggest that within this population, strategies over the past two decades to reduce complications from type 1 diabetes that cause cardiovascular disease and death are working, in principle. However, there is much need for further improvement. This includes the urgent need to understand why so few people with type 1 diabetes achieve good control of their blood sugar, and what can be done to improve this situation. It is also important to put more effort into keeping people with diabetes from taking up smoking or getting them to quit, as well as preventing them from getting overweight or promoting weight reduction, because this could further reduce the risks of cardiovascular disease and premature death.

**Additional Information:**

Please access these Web sites via the online version of this summary at http://dx.doi.org/10.1371/journal.pmed.1001321

- National Diabetes Information Clearinghouse, a service of the US National Institute of Diabetes and Digestive and Kidney Diseases, has information on heart disease and diabetes, on general complications of diabetes, and on the HbA_1c_ test (on this site and some others called A1C test) that measures control of blood sugar over the past 3 months
- Diabetes.co.uk provides general information on type 1 diabetes, its complications, and what people with the disease can do to reduce their risks
- The Canadian Diabetes Association offers a cardiovascular risk self-assessment tool and other relevant information
- The American Diabetes Association has information on the benefits and challenges of tight blood sugar control and how it is tested
- The Juvenile Diabetes Research Foundation funds research to prevent, cure, and treat type 1 diabetes
- Diabetes UK provides extensive information on diabetes for patients, carers, and clinicians

## Introduction

Type 1 diabetes (T1DM) is associated with an elevation in the risk of cardiovascular disease (CVD) and all-cause mortality [1]. Almost two decades ago the landmark Diabetes Care and Complications Trial (DCCT) demonstrated the preventability of many diabetic complications with tight glycaemic control [2] and longer term follow-up of the participants showed a reduction in CVD [3]. Since then guidelines have emphasised tighter glycaemic control as well as smoking cessation and blood pressure control. Above 40 y of age, statins are recommended for most patients [4],[5].

Whether these guidelines for management are now having an impact on the relative risks of CVD and mortality in those with T1DM is unclear, as contemporary nationwide data on risks relative to the non-diabetic population are sparse. Whilst several studies report CVD incidence among those with T1DM, there are few studies that have directly compared CVD incidence in T1DM with the general population [6] and most studies of mortality rates present long-term follow-up reflecting historical risks across the period of follow-up [7]–[9]. To obtain a comprehensive picture of the current relative CVD and mortality rates associated with T1DM we used a nationwide diabetes register from Scotland UK and data from the total non-diabetic population. To examine the scope for future reduction in relative risks we also examined achievement of current risk factor target levels.

## Methods

### Ethics Statement

Approval was obtained from the Scotland A Research Ethics Committee, Privacy (Caldicott) Guardians for the 14 Scottish Health Boards, and the Information Services Division (ISD) of National Health Service (NHS) Scotland Privacy Advisory Committee.

### Data Sources

In Scotland, primary and secondary health care is free in the NHS. Since 2000, a single nationwide clinical information system; the Scottish Care Information-Diabetes Collaboration (SCI-DC) database has captured registration of patients with T1DM.The registration occurs automatically when a patient is assigned a Read Code [10] for diabetes in a primary or secondary care health care information system. Since all but five of 1,076 general practices nationwide contribute data, it is estimated to capture over 99% of all patients nationally assigned a diagnostic Read Code for diabetes. From SCI-DC we extracted information on all people with T1DM aged ≥20 y who were alive anytime from 1st January 2005 to 31st May 2008. Thus, prevalent cases as of January 2005 (*n* = 19,161) and any incident cases of T1DM (*n* = 2,628) were included in the analysis. For the population of T1DM alive as of 31st May 2008 (the latest data available for research) we also extracted current risk factor (non-fasting lipids, blood pressure, current smoking, body mass index [BMI]) and prescribed medication (rather than encashed prescriptions) history. These data are uploaded into SCI-DC from all clinical encounters experienced by patients once registered. Risk factor data were not directly available for the general population but we provide comparisons with national surveys [11]. We defined T1DM on the basis of the type of diabetes assigned by the clinician but with the additional requirement that the prescription history not contradict this (i.e., no evidence of lengthy period of diabetes before insulin and no co-prescribing of non-metformin oral diabetes drugs).

We identified all major hospitalised CVD events for T1DM patients in 2005–2007 by linkage to the national hospital admissions data (the Scottish Morbidity Record SMR-01) held by the Information Services Division (ISD) of the NHS and death data provided by the National Records of Scotland (NRS). The SMR-01 captures all national public sector hospital admissions from 1981 onwards [12]. ISD also provided the counts of events and population denominators for the non-diabetic general population of Scotland aged ≥20 y for 2005–2007. CVD events were defined as hospital admissions or death with main/underlying cause with an ICD code for ischaemic/coronary heart disease (CHD) (ICD-9: 410–414, or ICD-10: I20–I25) or for cerebrovascular disease including transient cerebral ischaemic attacks and related syndromes (ICD-9: 430–438 or ICD-10: I60–I69 and G45). These ICD codes were chosen as they are used in the official national statistics for CVD. Since under ICD rules diabetes can be given as the underlying cause of death in certain situations even when an acute coronary event is present [13], we conducted a sensitivity analysis defining CVD deaths as those with the above CVD codes anywhere in the death certificate for those with diabetes as the underlying cause of death.

### Statistical Methods

Data for the total population were available in the form of counts of persons with an event in each calendar year, with the corresponding mid-year population estimates as an approximation of the person years, broken down by sex and age bands. To obtain counts of persons with events and denominators for the non-diabetic population we subtracted from the mid-year total population all those with any type of diabetes at any point in that year and we subtracted from the counts of persons with events for the total population all those with diabetes who had an event at any point in that year. This simplified approach means that a few months of person time pre-diabetes is also excluded for those with a diagnosis in the second half of the year. In practice the effect of this is negligible especially when one considers the arbitrariness of dates of diagnosis of type 2 diabetes. We chose to exclude all types of diabetes from the comparator group as it is the risk compared to a non-diabetic population that is of most clinical interest, to facilitate comparison with other studies and to ensure that changes in future estimates of IRRs are not confounded by changes in the prevalence or severity of type 2 diabetes. Inclusion of type 2 diabetes in the comparator group would be expected to reduce the IRRs. Individual level data on those with T1DM were grouped similarly to give counts of persons with events in each calendar year and the total person years observed within each calendar year. Incidence rate ratios (IRR) were estimated from a Poisson model with robust standard errors to allow for overdispersion. The IRRs associated with T1DM for a given attained age/sex group therefore represent the average effect of T1DM in that group across the 3 y of the study compared to those without any type of diabetes. IRR calculations were restricted to end December 2007 since partial year data for 2008 were not available for the non-diabetic population. All models adjust for a linear trend in calendar year, and age using 5-y age bands. We found significant interactions between sex and diabetes on the outcomes considered so we then analysed and have presented the data separately for men and women.

## Results

### Population Studied

During the period of study, between 2005 and 2007 inclusive, 26,026 people registered with T1DM were observed of whom 21,789 were ≥20 y old. The median duration of diabetes (interquartile range) was 17.5 y (9.3–27.0) in prevalent cases of T1DM at baseline. 20,668 of those had no CVD admission in the 10 y prior to start of follow-up. These people contributed 59,785 person years of observation for total mortality, 56,400 for first CVD event, and 57,060 for first CHD event. The non-diabetic population without a prior CVD event in the previous 10 y comprised 3.6 million people aged ≥20 and contributing 10.86 million person years of observation.

### CVD and Coronary Events

**Figure 1:**
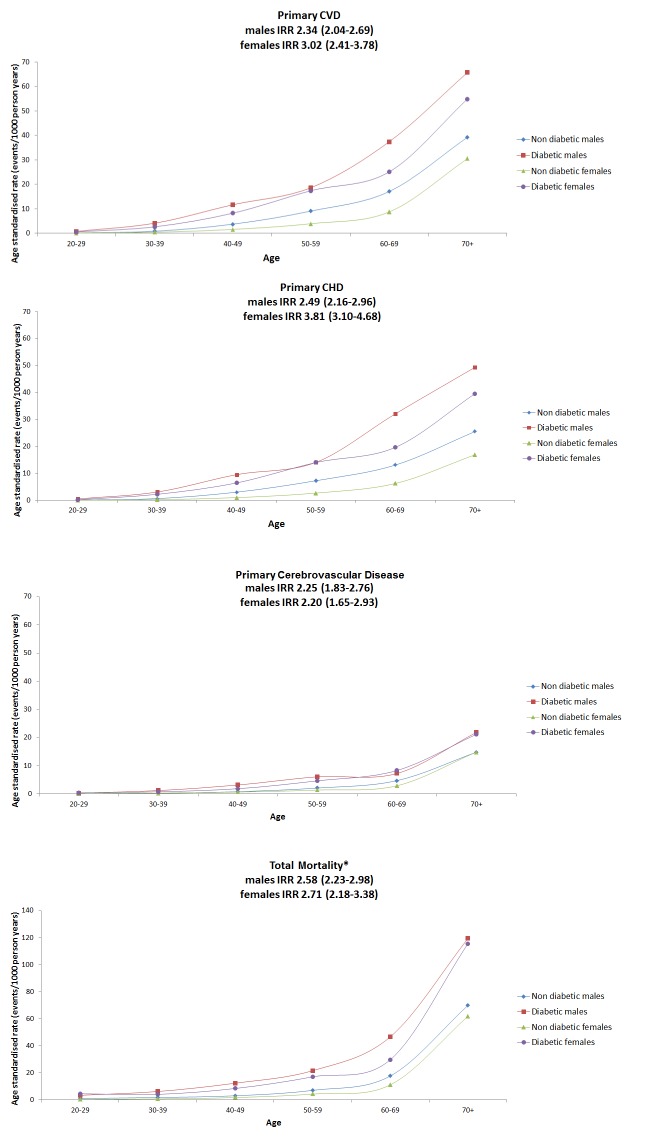
Age-standardised rates for primary CVD, primary CHD, primary cerebrovascular disease, and all-cause mortality by sex and age band for people with type 1 diabetes or non-diabetic in Scotland 2005–2007. All lines are interpolations. *y* axis for mortality panel has a different range to the other panels for purposes of display.

Table 1 shows the crude IRRs and the relative risks by age band for first major CVD events in those with T1DM compared to the non-diabetic population. Age-standardised rates are shown in Figure 1 with the lines shown being interpolations. Risk ratios were substantial, greater in women than men (*p* = 0.012 for the diabetes *x* sex interaction), and were highest in the younger age bands. Overall men with T1DM had an age-adjusted IRR of 2.3 (95% CI 2.0–2.7) and women with T1DM had an IRR of 3.0 (2.4–3.8) compared with the non-diabetic population. When CVD codes anywhere on the death record were considered as CVD deaths for those where diabetes was given as the underlying cause of death, then the IRR for first CVD event associated with T1DM was 2.5 (2.2–2.9) in men and 3.2 (2.6–3.9) in women. For first coronary events examined separately as with CVD, the IRR was higher in women with T1DM than men (Table S1). For first cerebrovascular events (Figure 1) the IRR was similar in men (2.3: 1.8–2.8) and women (2.2: 1.7–2.9) with T1DM. The grouped data on the non-diabetic population for cerebrovascular events include transient ischaemic attacks (TIAs) and therefore these have been included for the T1DM population also. If hypoglycaemic episodes for example were miscoded as TIAs in those with T1DM this could inflate the IRRs for cerebrovascular events associated with diabetes. However, even in an extreme sensitivity analysis where we exclude all TIAs in the T1DM population only, the IRRs for cerebrovascular events remained substantially elevated at 2.06 (1.69–2.51) in men and 1.89 (1.38–2.58) in women.

**Table 1:** Incidence rates and IRRs of first cardiovascular event in those with type 1 diabetes compared with the non-diabetic population.

Sex, Age (y)	Events	Person Years	Crude Rate per 1,000 Person Years (SE)	Events	Person Years	Crude Rate per 1,000 Person Years (SE)	Age-Adjusted IRR	95% CI
	Type 1 Population			Non-diabetic Population				
**Men**								
All ages^a^	393	31,568	12.45 (0.63)	43,514	5,075,885	8.57 (0.04)	2.34	(2.04–2.69)
20–39	39	14,286	2.73 (0.44)	1026	1,974,234	0.52 (0.02)	4.80	(3.73–6.18)
40–49	99	8,579	11.54 (1.16)	4,070	1,085,745	3.75 (0.06)	3.11	(2.69–3.59)
50–59	97	5,215	18.60 (1.89)	8,138	893,125	9.11 (0.10)	2.10	(1.63–2.70)
60–69	86	2,335	36.83 (3.97)	10,629	621,951	17.09 (0.17)	2.19	(1.75–2.74)
70+	72	1,153	62.43 (7.36)	19,651	500,830	39.24 (0.28)	1.71	(1.28–2.29)
**Women**								
All ages	259	24,832	10.43 (0.65)	39,202	5,788,221	6.77 (0.03)	3.02	(2.41–3.78)
20–39	20	11,353	1.76 (0.39)	634	2,036,321	0.31 (0.01)	5.48	(4.19–7.16)
40–49	51	6,266	8.14 (1.14)	1,918	1,181,395	1.62 (0.04)	5.06	(3.78–6.78)
50–59	64	3,725	17.18 (2.15)	3,831	968,633	3.96 (0.06)	4.47	(3.92–5.10)
60–69	50	2,038	24.53 (3.47)	6,453	738,632	8.74 (0.11)	2.83	(2.27–3.53)
70+	74	1,450	51.02 (5.93)	26,366	863,240	30.54 (0.19)	1.85	(1.44–2.37)

SE, standard error.

a All those aged ≥20 y and observed in the period 2005–2007.

The IRR for CVD mortality associated with T1DM was similar in men at 3.4 (2.7–4.2) as in women at 3.5 (2.4–4.9). When CVD codes anywhere on the death record were considered as CVD deaths for those where diabetes was given as the underlying cause of death then the IRR for CVD mortality was higher in both sexes at 4.5 (3.7–5.6) in men and 4.4 (3.1–6.3) in women.

As it has often been asserted that the increased risk of CVD in diabetes is confined to those with renal impairment we examined risks by estimated glomerular filtration rate (eGFR). When stratified by eGFR, the IRR for CVD associated with T1DM adjusted for age was 7.06 (95% CI 5.04–9.89), 3.13 (95% CI 2.43–4.05), and 1.83 (95% CI 1.57–2.13) in those with an eGFR <30, 30–59, and ≥60 ml/min/1.73 m^2^, respectively, in men and 10.92 (95% CI 7.87–15.16), 2.51 (1.78–3.54), and 2.55 (95% CI 2.06–3.16) in women. Among the subset of individuals with T1DM with an eGFR >60 ml/min/1.73 m^2^ in whom the exact eGFR was known, the IRR for CVD for those 8,848 individuals with an eGFR >90 ml/min/1.73 m^2^ was 2.13 (95% CI 1.65–2.74) in men and 3.69 (95% CI 2.44–5.57) in women.

### All-Cause Mortality

**Figure 2:**
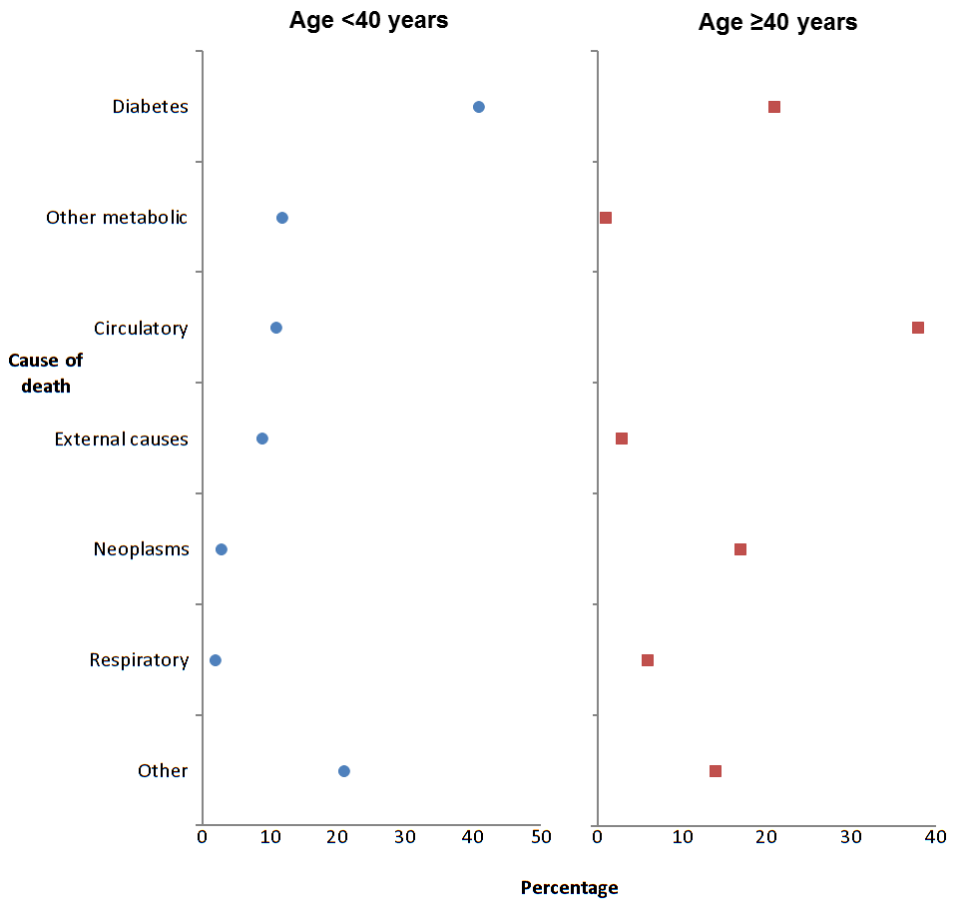
Most common underlying causes of death in type 1 diabetes, 2005–2007.

Figure 1 and Table 2 show the age-standardised rates of all-cause mortality by age bands in those with and without diabetes, by sex. The IRR for all-cause mortality associated with T1DM was similar in men at 2.6 (95% CI 2.2–3.0, *p*<0.001) and women at 2.7 (2.2–3.4, *p*<0.001) and decreased with age. Of the 123 deaths in 10,173 people with T1DM aged <40 y in any of the years 2005–2007 (absolute rate 4.8/1,000 person years at risk), the top three underlying causes were diabetes mellitus (41.4%; of which coma or ketoacidosis accounted for 34 of 51 deaths), other metabolic disorders (12.2%; 15 deaths), and circulatory disease (11.4%; 14 deaths). Of the 907 deaths in the 12,729 with T1DM age ≥40 y (absolute rate 26.7/1,000 person years at risk), the leading causes were circulatory disease (38.5%; 349 deaths), diabetes mellitus (20.6%; of which coma and ketoacidosis accounted for 37 and renal complications 47 of 187 deaths), and neoplasm (17.0%; 154 deaths) (Figure 2). Overall 63% of death certificates in those <40 y and 69% in those ≥40 y mentioned diabetes. The age band-specific crude rates shown in Tables 1 and 2 can be used to estimate the absolute risks difference between those with and without T1DM for a given age. For example, at the attained age of 60–69 y there are approximately three extra deaths per 100 per year in men (28.51/1,000 person years at risk), and two per 100 per year for women (17.99/1,000 person years at risk) with TIDM. Mortality from all causes other than diabetes and CVD was also increased at IRR 1.79 (95% CI 1.57–2.04) in men and 1.93 (95% CI 1.62–2.30) in women overall.

**Table 2:** Incidence rates and IRRs for total mortality in those with type 1 diabetes compared with the non-diabetic population.

Sex, Age (y)	Events Person Years		Crude Rate per 1,000 Person Years (SE)	Events Person Years		Crude Rate per 1,000 Person Years (SE)	IRR	95% CI
	Type 1 Population			Non-diabetic Population				
**Men**								
All ages^a^	607	33,583	18.07 (0.73)	67,411	5,287,038	12.8 (0.05)	2.58	(2.23–2.98)
20–29 y	21	6,103	3.44 (0.75)	1,071	976,523	1.1 (0.03)	3.14	(2.36–4.18)
30–39 y	52	8,292	6.27 (0.87)	1,851	1,001,013	1.8 (0.04)	3.39	(2.84–4.04)
40–49 y	111	8,979	12.36 (1.17)	3,428	1,101,013	3.1 (0.05)	3.99	(3.82–4.15)
50–69 y	124	5,795	21.40 (1.92)	6,641	930,825	7.1 (0.09)	3.09	(2.73–3.50)
60–69 y	133	2,878	46.21 (4.01)	11,879	670,964	17.7 (0.16)	2.67	(2.30–3.09)
70 plus	166	1,536	108.05 (8.39)	42,541	606,700	70.1 (0.34)	1.75	(1.56–1.96)
**Women**								
All ages^a^	423	26,202	16.14 (0.78)	76,222	5,952,362	12.81 (0.05)	2.71	(2.18–3.38)
20–29 y	23	4,952	4.64 (0.97)	332	961,843	0.35 (0.02)	13.46	(10.03–18.06)
30–39 y	27	6,462	4.18 (0.80)	874	1,077,062	0.81 (0.03)	5.16	(3.69–7.23)
40–49 y	55	6,524	8.43 (1.14)	2,139	1,189,860	1.80 (0.04)	4.72	(3.62–6.15)
50–69 y	69	4,105	16.81 (2.02)	4,352	988,915	4.40 (0.06)	3.93	(3.41–4.53)
60–69 y	70	2,400	29.17 (3.49)	8,583	767,621	11.18 (0.12)	2.65	(2.11–3.32)
70 plus	179	1,759	101.78 (7.61)	59,942	967,061	61.98 (0.25)	1.92	(1.67–2.21)

a All those aged ≥20 and observed in the period 2005–2007.

SE, standard error.

### Effect of Diabetes Duration

The IRRs for CVD and for total mortality associated with T1DM varied by tertile of diabetes duration, adjusted for age, though they were high even in those with shortest duration. For CVD the IRRs were 2.17 (95% CI 1.69–2.77), 2.37 (95% CI 1.98–2.83), and 2.41 (2.01–2.88) in those with duration <10.8, 10.8–22, and ≥22.0 y, respectively, in men, and 2.63 (95% CI 1.95–3.54), 2.91 (95% CI 2.05–4.13), and 3.22 (95% CI 2.52–4.13) in women adjusted for age. For total mortality the IRRs were 1.67 (95% CI 1.25–2.24), 2.11 (95% CI 1.71–2.60), and 2.11 (95% CI 1.60–2.79) in those with duration <10.8, 10.8–22, and ≥22.0 y, respectively, in men, and 1.62 (95% CI 1.12–2.33), 1.87 (95% CI 1.18–2.97), and 2.09 (95% CI 1.44–3.04) in women adjusted for age.

### Risk Factor Control in Those with Type 1 Diabetes

**Figure 3:**
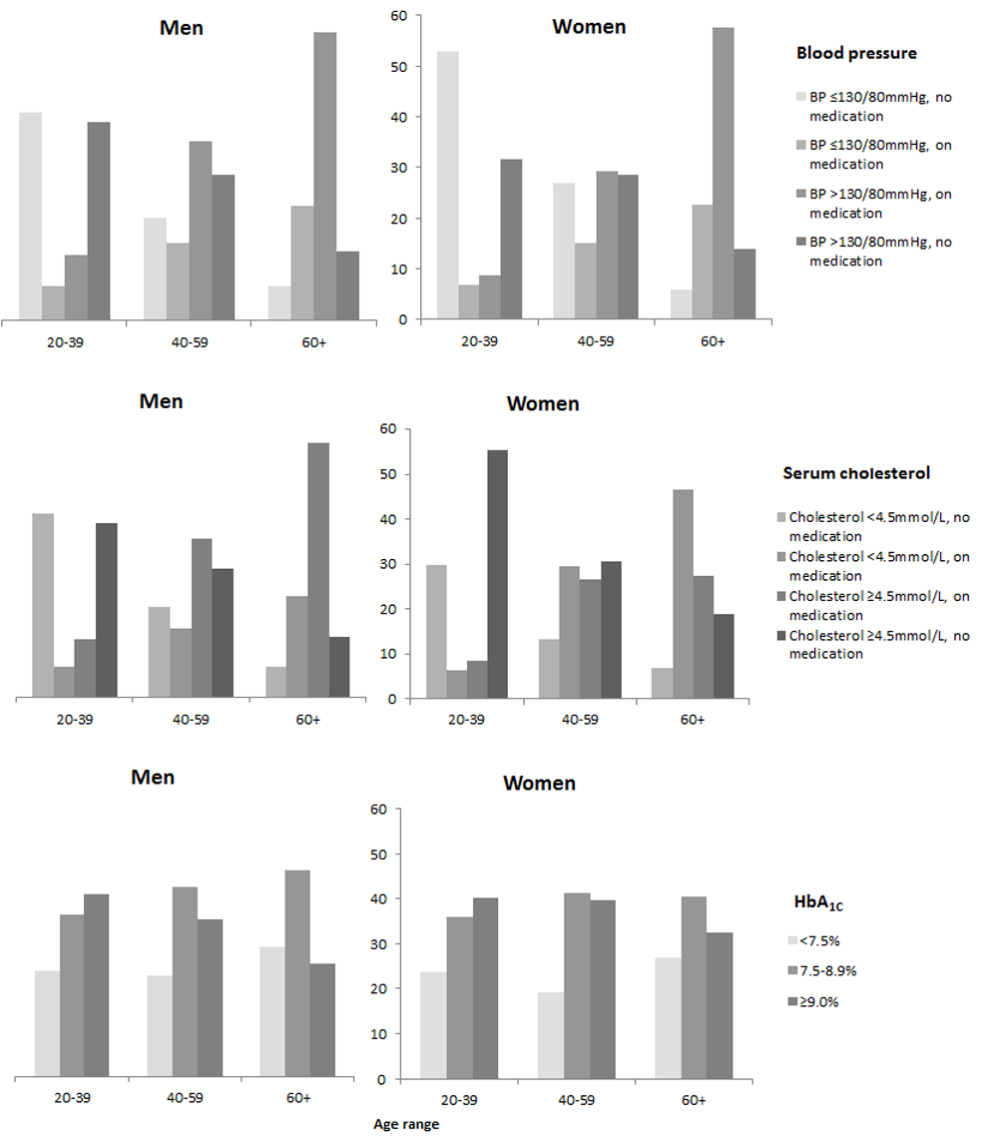
Risk factor prevalence in type 1 diabetes, May 2008.

Figure 3 and Table 3 show risk factor rates and the extent to which the main targets of therapy were achieved as of 31st May 2008. We did not have data on risk factors in the non-diabetic population but Table S2 shows simple comparisons with the published data from the Scottish Health Survey. Of note, the median HbA_1c_ (8.4 in men, 8.5 in women) was very far from the targets that vary between 7% and 7.5% in international guidelines (Table 3). Overall only 13% achieved target HbA_1c_ of <7%, 23% an HbA_1c_ of <7.5%, and 37% had very poor (≥9%) glycaemic control. 30% of men and 25% of women with T1DM were current smokers. As shown in Table S2, smoking rates in men with T1DM were similar to the general population and were only slightly lower in women with T1DM. Median BMI was 27 kg/m^2^ in men and women with T1DM. Overall obesity rates were slightly lower than the general population rates in T1DM men but similar in T1DM women (Table S2). Examined by age group (unpublished data) obesity rates were slightly higher in those with T1DM <55 y of age and then lower thereafter. The Scottish Intercollegiate Guidelines Network for Diabetes [5] recommend achieving a systolic blood pressure (BP) <130 mmHg and a diastolic BP ≤80 mmHg. These cut-offs were used to define hypertension in Figure 3. Overall 73% of men and 66% of women with T1DM either had a raised blood pressure using the 130/80 mmHg threshold or were on anti-hypertensive medication. Of these, 82% of men and 80% of women had BP readings above the threshold such that overall 60% of men and 53% of women with T1DM had a blood pressure above the target of 130/80 mmHg. In comparison with the general population, hypertension rates in men and women with T1DM were higher, but treatment and control rates were also higher (Table S2).

**Table 3:** Risk factor levels in all those with type 1 diabetes aged ≥20 by age and sex at most recent assessment.

Age, y	Men				Women				Both Sexes Total *n* = 21,290^a^
	20–39	40–59	60+	Total	20–39	40–59	60+	Total	
	*n* = 5,217	*n* = 5,260	*n* = 1,537	*n* = 12,014	*n* = 4,060	*n* = 3,789	*n* = 1,427	*n* = 9,276	
Diabetes duration, y	12.9 (6.4–20.4)	22.4 (13.4–31.4)	31.0 (18.4–41.4)	18.4 (9.4–28.4)	14.4 (7.6–21.4)	24.4 (15.3–33.0)	30.4 (16.6–42.4)	19.4 (11.2–29.4)	18.5 (10.4–28.4)
Systolic BP, mmHg	128 (119–137)	132 (122–142)	137 (126–147)	130 (120–140)	121 (111–131)	130 (120–140)	138 (127–148)	128 (116–140)	130 (120–140)
Diastolic BP, mmHg	76 (70–81)	77 (70–82)	71 (64–79)	76 (70–81)	75 (68–80)	74 (68–80)	70 (63–78)	74 (67–80)	75 (68–80)
Total cholesterol, mmol/l	4.6 (4.0–5.3)	4.4 (3.8–5.1)	4.0 (3.5–4.6)	4.4 (3.9–5.1)	4.8 (4.2–5.4)	4.6 (4.0–5.2)	4.4 (3.9–5.0)	4.6 (4.1–5.3)	4.5 (3.9–5.2)
Triglyceride, mmol/l	1.3 (0.9–2.0)	1.2 (0.9–1.8)	1.2 (0.8–1.7)	1.2 (0.9–1.9)	1.1 (0.8–1.7)	1.0 (0.7–1.5)	1.1 (0.8–1.6)	1.1 (0.8–1.6)	1.2 (0.8–1.7)
HDL cholesterol, mmol/l	1.3 (1.1–1.6)	1.4 (1.1–1.7)	1.4 (1.1–1.7)	1.4 (1.1–1.6)	1.5 (1.3–1.8)	1.7 (1.3–2.0)	1.7 (1.4–2.1)	1.6 (1.3–1.9)	1.5 (1.2–1.8)
BMI, kg/m^2^	25.7 (23.1–29.0)	27.3 (24.6–30.2)	27.1 (24.3–30.1)	26.6 (23.9–29.7)	26.2 (23.4–30.1)	27.0 (23.9–31.3)	26.8 (23.6–30.7)	26.6 (23.6–30.7)	26.6 (23.8–30.1)
HbA_1c_, %	8.6 (7.5–9.7)	8.4 (7.5–9.4)	8.1 (7.3–9.0)	8.4 (7.5–9.5)	8.5 (7.5–9.8)	8.6 (7.7–9.6)	8.3 (7.4–9.3)	8.5 (7.6–9.6)	8.5 (7.5–9.5)
Current smoker	33.2 (0.68)	29.9 (0.65)	19.1 (1.02)	29.9 (0.43)	25.9 (0.70)	26.8 (0.73)	15.4 (0.98)	24.6 (0.46)	27.6 (0.31)
On regular aspirin	6.4 (0.35)	36.2 (0.68)	59.9 (1.28)	26.6 (0.42)	4.9 (0.35)	29.8 (0.76)	54.2 (1.36)	22.9 (0.45)	25.0 (0.31)
On a statin	17.3 (0.55)	58.8 (0.70)	72.8 (1.16)	43.0 (0.47)	13.4 (0.56)	54.7 (0.83)	73.6 (1.20)	39.9 (0.53)	41.7 (0.35)
On anti-hypertensive medication	18.5 (0.56)	49.7 (0.71)	79.5 (1.06)	40.3 (0.46)	15.1 (0.59)	43.5 (0.83)	79.4 (1.10)	36.9 (0.52)	38.9 (0.35)
Of treated, those on an ACE inhibitor	80.2 (1.34)	76.1 (0.86)	70.8 (1.33)	75.5 (0.64)	66.7 (1.99)	65.3 (1.20)	60.9 (1.50)	64.1 (0.85)	70.8 (0.52)

Values are median (25th–75th percentile) for continuous variables, % (standard error) for categorical variables.

a Numbers varied slightly for available data for different risk factors.

The Scottish Intercollegiate Guidelines Network for Diabetes [5] recommend consideration of statin therapy in all patients with T1DM aged ≥40 y and other guidelines give various targets for total cholesterol between 3.4 and 4.5 mmol/l [14]. As shown in Figure 3 and Table 3, statin therapy rose steeply with age so that median cholesterol was lower with older age but overall 39% of those aged ≥40 y were not on statin therapy. The median total cholesterol was 4.5 mmol/l with 25% having a total cholesterol ≥5.2 mmol/l. Compared with the general population, however, elevated total cholesterol levels were substantially lower in those with T1DM (Table S2).

## Discussion

The data presented provide a nationwide analysis of the prevailing risk factor levels in people with T1DM and associated contemporary CVD and mortality risks. A valuable aspect of this study is that the large sample size and comprehensive capture of those with T1DM in Scotland means these high risks and risk factor levels are truly representative and without selection bias. The large sample size has allowed us to provide precise estimates of current risks. The data demonstrate the following key clinical points.

First, the risks we report are substantially lower than those found in studies that covered earlier decades, suggesting that strategies to reduce complications of diabetes are working. Second, despite these reductions the relative risk of CVD, CHD, stroke and all-cause mortality continue to be unacceptably high for this patient population. For example at the attained age of 60–69 y, there are approximately three extra deaths per 100 per year in men (28.51/1,000 person years at risk), and two per 100 per year for women (17.99/1,000 person years at risk) with T1DM. As expected the elevation in CVD risk is highest in those with renal impairment but there is still a substantial elevation in risk when eGFR is not reduced. Whilst CVD remains the single largest category of deaths in those aged ≥40 y, these data also emphasise that mortality from causes other than CVD and diabetes are also elevated in diabetes showing the multisystem nature of complications of this disease. Third, of particular concern is the high number of deaths in those aged <40 y that are due to diabetic ketoacidosis or coma (ICD10 codes do not differentiate hypo- and hyperglycaemic coma). Fourth, it is now 18 y since the Diabetes Control and Complications Trial (DCCT) trial showed the benefits of achieving an HbA_1c_ <7% [2]. However such levels remain a very distant target for the majority of patients with T1DM, indicating that we need to really re-think strategies for improving HbA_1c_. Fifth, there is substantial scope for much more control of risk factors for diabetic complications including an assertive attempt at preventing smoking uptake in those with T1DM. Whilst further research into the pathogenesis of diabetic complications is warranted, a major research priority should be understanding the barriers to applying what we already know, i.e., achieving risk factor control. In particular there is substantial scope for reducing smoking rates. We found similar smoking rates in the type 1 compared to background population (Table S2) [11]. Direct comparisons within population of smoking rates in T1DM with non-diabetic persons are sparse. In Germany similar rates were reported in young adults with T1DM to our rates in young adults. German background smoking rates are similar to that for Scotland at 26% overall with highest rates being in young adults [15],[16]. In the US background smoking rates are lower than in Scotland at 18% current smoking in adults. Current data from the behavioural Risk Factor Surveillance system [17] show this lower US prevalence is true for those with and without diabetes. However those with diabetes aged 18–24 y (who will mostly have T1DM) have slightly higher rates of smoking (29%) compared with those without diabetes at this age (22%).

A strength of our data is that the risks we report reflect the current relative risks given the mix of duration of diabetes (and survival until recently) and current mix of attained ages pertaining in the population here and now. Such contemporary estimates are essential as a baseline for assessing impact of future changes in management and provide the context for research into CVD in T1DM in the future. In contrast long-term follow-up of cohorts has provided useful historical estimates of risks, the summary estimates from which are determined by the relative risks pertaining right across the time period of follow-up. Furthermore many studies with longer term follow-up have included only those below a certain age at baseline so that the overall risks pertain only to that fraction of those with T1DM below a certain attained age. As we have shown the relative risks vary very widely with age band so these differences in inclusion criteria make comparisons between studies difficult. However, even allowing for differences in inclusion criteria and definitions of CVD between studies, our data show substantially lower relative risks for CVD pertaining now, particularly for women, than have been reported in such previous studies with longer term follow-up [1]. For example in the Wisconsin Epidemiologic Study of Diabetic Retinopathy (WESDR) for the period 1980–1988, Moss et al. reported standardised mortality ratios (SMRs) for CHD of 9.1 in males and 13.5 in females in 1,300 young onset diabetes patients [8]. In the 1986 National Mortality Follow Back Survey in the US, CHD mortality rates in those with diabetes <55 y were 8-fold in men and 16-fold in women compared with the general population [18] . Laing et al. reported CHD SMRs of 4.5 and 8.8 in men and women, respectively, relative to the general population for a period of follow-up 1972–2000, with SMRs as high as 8.9 and 41.7 in men and women, respectively, between ages 1–40 y [19]. A Norwegian cohort with long-term follow-up reported SMRs for CVD of 11 in men and 10 for women but the maximum attainable age at follow-up was 42 y and the total number of events was 14 [20]. In a recent long-term follow-up (1970–2007) of a Finnish cohort the SMR for CHD was 17.4 in those with diabetes onset below age 15 y, but estimates specifically for recent time periods were not shown [21]. In the Allegheny County cohort long-term follow-up (1965–2008), SMRs for CVD were 9 in men and 25 in women with a mean age at follow-up of 51 y [22]. In contrast our CVD mortality ratios estimated for all ages between 2005–2007 were lower at 3.4 in men and 3.5 in women. Studies that directly compare T1DM CVD or CHD incidence, as distinct from mortality, with the general population are sparse; in a 7-y follow-up of the General Practice Research Database for the more recent period 1990–1999 the relative risk for CHD incidence was 3.0 (2.2–4.1) in men and was 7.6 (4.9–12.0) in women with T1DM [6]. These risks compare with CHD incidence relative risks of 2.5 (2.2–3.0) in men and 3.8 (3.1–4.7) in women in our study. It is difficult to definitively separate out calendar period effects in making comparisons between our data and these other studies, but it is very likely that the differences partly reflect improvement in CVD relative risks over the longer term with the extent of recent changes being less certain. It would be of interest to examine short term current CVD rate ratios in these other cohorts as we have done. Some of the above studies have compared risks with the general population, including all those with diabetes, as distinct from the specifically non-diabetic population as we have done. However comparisons with the general population should show smaller relative risks than comparisons with the non-diabetic population so this cannot explain the lower relative risks we observe than in previous studies.

Our data suggest that there has also been some improvement in relative total mortality over the preceding decades but the extent of recent changes is less certain. In the WESDR study (*n* = 1,200) for 1980–1988 the SMR for total mortality was 7 in males and 9 in females [8]. Follow-up of the Allegheny County cohort (*n* = 1,043) from 1965–2008 reported SMRs of 5 in men and 13 in women with clear downward trend through time [7]. In one of the largest previous studies with 13-y average follow-up ending in 1997 the SMR was 2.7 (2.5–2.9) in men and 4.0 (3.6–4.4) in women [23]. An analysis of total mortality from Finland covering 1970–2007 showed that relative mortality has declined for younger onset T1DM patients but surprisingly increased in older onset type 1 patients, with an overall SMR of 3.6 and 2.8 in these two cohorts across the period [21]. In the General Practice Research Database study for 1990–1999 the relative mortality risks were 3.3 (95% CI 2.7–4.0) in men and 4.5 (95% CI 3.5–5.6) in women [9]. These data compare with lower relative risks for mortality of 2.6 (2.2–3.0) in men and 2.7 (2.2–3.4) in women in our study.

Mean HbA_1c_ in the Pittsburgh Epidemiology of Diabetes Complications (EDC) was 10.3% considerably higher than the median of 8.4% for men and 8.5% for women that we report [24], but our results compare with findings elsewhere in Europe and Australia [25],[26]. These observations suggest that in most health care situations maintenance of tight glycaemic control is extremely difficult to achieve in the majority of T1DM patients. Blood pressure control was considerably poorer that that seen in other reports from the UK [26],[27] and the EURODIAB PCS [28] and FinnDiane cohorts [29]. In contrast, median cholesterol values were close to ESC/EASD recommended levels [14], and lower than those seen in comparable studies across Europe [27]–[30].

We report, consistent with previous studies, that the relative risk for CVD and CHD events was greater for women than men [6],[31]. It is not clear why relatively speaking T1DM affects CVD risk more in women than men, or in other words that the sex difference in CVD found in the non-diabetic population is narrowed in T1DM. Previous work suggests that the greater relative risk in women is not explained by a more adverse known CVD risk factor profile for women than men with T1DM [31], though we found a more favourable difference in BMI and total cholesterol levels in T1DM men than women relative to the general population. These greater risks for events in women than men with T1DM are not found when fatal CVD events alone are examined. This finding could be explained either by a diagnostic bias whereby admissions are more likely to be classified as due to CVD in women than men or CVD deaths being less likely to be classified as due to CVD in women. Alternatively perhaps more effective treatment reduces the case fatality more in women than men. Some limitations of our analysis are that since the establishment of the diabetes register is relatively recent we cannot report time trends in risk ratios. Another limitation is that we did not have individual level data on events and risk factors in the non-diabetic population. While our data are quite contemporary in comparison with many published analyses, any further improvement in risk factor control, including statin usage, in the past 5 y might be expected to reduce current rates even further, emphasising the need for ongoing monitoring of IRRs for improvements.

A striking feature of the data is the very low rate of achievement of glycaemic control targets. The need for improved provision of structured patient education to enable self-management strategies has been emphasised [5],[14]. Increased patient education may have been available to the minority of patients in the study period but it is currently being expanded across the UK. The role of continuous subcutaneous insulin infusion (CSII) in improving overall glycaemic control remains controversial. Whilst we did not have individual level data on insulin regime or pump usage we know that currently only 2.5% of patients in the Scottish population receive CSII therapy [32]. This number is lower than even conservative guidelines on CSII usage, but a recently announced increase in provision of CSII [33] is likely to improve HbA_1c_ for some patients. However our data emphasise the need for more adjunctive therapies beyond insulin to help patients achieve better control whilst maintaining quality of life and avoiding hypoglycaemia. We are currently investigating metformin as one such therapy in the Juvenile Diabetes Research Foundation (JDRF)-funded REMOVAL trial [34]. Other important trials of risk reduction in T1DM include the ongoing AdDIT trial of statin therapy in teenagers with diabetes [35]. Finally, whilst here we provide data on crude rates of CVD by age, clinicians need better data on absolute risk of CVD for different combinations of risk factors for patients with T1DM, i.e., a risk engine, to tailor more intensive management and early statin therapy to those most at risk. This area is the focus of our ongoing work.

## Supporting Information

Table S1 Incidence rates and IRR of first CHD event in those with type 1 diabetes compared with the non-diabetic population.
(DOCX)

Table S2 Hypertension and raised cholesterol in population with type 1 diabetes and general population [11].
(DOCX)

## Abbreviations

BMI: body mass index
BP: blood pressure
CHD: coronary heart disease
CVD: cardiovascular disease
eGFR: estimated glomerular filtration rate
IRR: incidence rate ratio
SMR: standardised mortality ratio
T1DM: type 1 diabetes
