# Supplementary material for: Risk of Cardiovascular Disease and Total Mortality in Adults with Type 1 Diabetes: Scottish Registry Linkage Study
Source: PLoS Med. 2012 Oct 2;9(10):e1001321. doi: 10.1371/journal.pmed.1001321 (PMC3462745; doi:10.1371/journal.pmed.1001321)
Supplement: Table S1 — Incidence rates and IRR of first CHD event in those with type 1 diabetes compared with the non-diabetic population. (DOCX) [file pmed.1001321.s001.docx]

**Supplementary Table 1. Incidence Rates And Incidence Rate Ratio Of First Coronary Heart Disease Event In Those With Type 1 Diabetes Compared With The Non-Diabetic Population**

|  | **Events** | **Person years** | **Crude Rate per 1000 person years (SE)** | **Events** | **Person years** | **Crude Rate per 1000 person years (SE)** | **Age-adjusted incidence rate ratio** | **95% CI** |
| --- | --- | --- | --- | --- | --- | --- | --- | --- |
| **Men** | **Type 1 population** | |  | **Non-diabetic population** | |  |  |  |
| All ages* | 321 | 31,962 | 10.04 (0.56) | 32,200 | 5,127,420 | 6.28 (0.04) | 2.49 | (2.16–2.96) |
| 20-39 years | 28 | 14,320 | 1.96 (0.37) | 657 | 1,975,994 | 0.33 (0.01) | 5.30 | (3.80–7.40) |
| 40-49 years | 81 | 8,657 | 9.36 (1.04) | 3,246 | 1,089,281 | 2.98 (0.05) | 3.17 | (2.74–3.67) |
| 50-69 years | 77 | 5,342 | 14.41 (1.64) | 6,508 | 899,713 | 7.23 (0.09) | 2.05 | (1.47–2.85) |
| 60-69 years | 77 | 2,415 | 31.89 (3.63) | 8,239 | 631,199 | 13.05 (0.14) | 2.48 | (1.95–3.17) |
| 70 plus | 58 | 1,228 | 47.22 (6.20) | 13,550 | 531,233 | 25.51 (0.22) | 1.98 | (1.60–2.44) |
| **Women** |  | |  |  | |  |  |  |
| All ages* | 205 | 25,098 | 8.17 (0.57) | 23,815 | 5,846,057 | 4.07 (0.03) | 3.81 | (3.10–4.68) |
| 20-39 years | 15 | 11,365 | 1.32 (0.34) | 227 | 2,038,242 | 0.11 (0.01) | 11.33 | (7.89–16.26) |
| 40-49 years | 40 | 6,309 | 6.34 (1.00) | 1,148 | 1,184,961 | 0.97 (0.03) | 6.61 | (4.77–9.17) |
| 50-69 years | 53 | 3,810 | 13.91 (1.91) | 2,584 | 974,806 | 2.65 (0.05) | 5.40 | (4.47–6.53) |
| 60-69 years | 40 | 2,095 | 19.09 (3.02) | 4,628 | 745,729 | 6.21 (0.09) | 3.11 | (2.45–3.94) |
| 70 plus | 57 | 1,519 | 37.54 (4.97) | 15,228 | 902,319 | 16.88 (0.14) | 2.41 | (2.13–2.73) |

*All those aged 20 plus without prior events and observed in the period 2005 to 2007.
