# Supplementary material for: Risk of Cardiovascular Disease and Total Mortality in Adults with Type 1 Diabetes: Scottish Registry Linkage Study
Source: PLoS Med. 2012 Oct 2;9(10):e1001321. doi: 10.1371/journal.pmed.1001321 (PMC3462745; doi:10.1371/journal.pmed.1001321)
Supplement: Table S2 — Hypertension and raised cholesterol in population with type 1 diabetes and general population [11]. (DOCX) [file pmed.1001321.s002.docx]

**Supplementary Table 2. Hypertension And Raised Cholesterol In Population With Type 1 Diabetes And General Population. [11]**

|  |  |  | **Male** | **Female** |
| --- | --- | --- | --- | --- |
|  |  |  | **Total 16+** | **Total 16+** |
| BP >140/90mmHg, or on BP Drugs (%) | T1DM |  | 53.9 | 47.1 |
|  | General Population | | 34.6 | 30.4 |
| (%) of these on treatment | T1DM |  | 74.0 | 75.7 |
|  | General Population | | 41.9 | 53.0 |
| (%) BP controlled | T1DM | | 43.5 | 45.2 |
|  | General Population | | 24.0 | 27.0 |
| Current smoker (%) | T1DM |  | 29.1 | 23.9 |
|  | General Population | | 26.0 | 25.0 |
| BMI ≥30 (%) | T1DM |  | 22.8 | 27.5 |
|  | General Population | | 26.9 | 27.6 |
| Cholesterol ≥5mmol/l (%)* | T1DM | | 30.5 | 37.8 |
|  | General Population | | 63.8 | 62.7 |

Note that to allow comparison with the general population data the age range begins at 16 years rather than 20 as in the main tables

*Cholesterol data is for age range 16-64 to compare with available population data
